# A Novel Method to Predict Carbohydrate and Energy Expenditure During Endurance Exercise Using Measures of Training Load

**DOI:** 10.1007/s40279-024-02131-z

**Published:** 2024-11-01

**Authors:** Jeffrey A. Rothschild, Stuart Hofmeyr, Shaun J. McLaren, Ed Maunder

**Affiliations:** 1 High Performance Sport New Zealand (HPSNZ), 17 Antares Place, Mairangi Bay, Auckland, 0632 New Zealand; 2 Sports Performance Research Institute New Zealand (SPRINZ), Auckland University of Technology, Auckland, New Zealand; 3 Newcastle Falcons Rugby Club, Newcastle upon Tyne, UK; 4 Department of Sport and Exercise Sciences, Manchester Metropolitan University Institute of Sport, Manchester, UK

## Abstract

**Background:**

Sports nutrition guidelines recommend carbohydrate (CHO) intake be individualized to the athlete and modulated according to changes in training load. However, there are limited methods to assess CHO utilization during training sessions.

**Objectives:**

We aimed to (1) quantify bivariate relationships between both CHO and overall energy expenditure (EE) during exercise and commonly used, non-invasive measures of training load across sessions of varying duration and intensity and (2) build and evaluate prediction models to estimate CHO utilization and EE with the same training load measures and easily quantified individual factors.

**Methods:**

This study was undertaken in two parts: a primary study, where participants performed four different laboratory-based cycle training sessions, and a validation study where different participants performed a single laboratory-based training session using one of three exercise modalities (cycling, running, or kayaking). The primary study included 15 cyclists (five female; maximal oxygen consumption [$\dot{V}$O_2_max], 51.9 ± 7.2 mL/kg/min), the validation study included 21 cyclists (seven female; $\dot{V}$O_2_max 53.5 ± 11.0 mL/kg/min), 20 runners (six female; $\dot{V}$O_2_max 57.5 ± 7.2 mL/kg/min), and 18 kayakers (five female; $\dot{V}$O_2_max 45.6 ± 4.8 mL/kg/min). Training sessions were quantified using six training load metrics: two using heart rate, three using power, and one using perceived exertion. Carbohydrate use and EE were determined separately for aerobic (gas exchange) and anaerobic (net lactate accumulation, body mass, and O_2_ lactate equivalent method) energy systems and summed. Repeated-measures correlations were used to examine relationships between training load and both CHO utilization and EE. General estimating equations were used to model CHO utilization and EE, using training load alongside measures of fitness and sex. Models were built in the primary study and tested in the validation study. Model performance is reported as the coefficient of determination (*R*^2^) and mean absolute error, with measures of calibration used for model evaluation and for sport-specific model re-calibration.

**Results:**

Very-large to near-perfect within-subject correlations (*r* = 0.76–0.96) were evident between all training load metrics and both CHO utilization and EE. In the primary study, all models explained a large amount of variance (*R*^2^ = 0.77–0.96) and displayed good accuracy (mean absolute error; CHO = 16–21 g [10–14%], EE = 53–82 kcal [7–11%]). In the validation study, the mean absolute error ranged from 16–50 g [15–45%] for CHO models to 53–182 kcal [9–31%] for EE models. The calibrated mean absolute error ranged from 9–20 g [8–18%] for CHO models to 36–72 kcal [6–12%] for EE models.

**Conclusions:**

At the individual level, there are strong linear relationships between all measures of training load and both CHO utilization and EE during cycling. When combined with other measures of fitness, EE and CHO utilization during cycling can be estimated accurately. These models can be applied in running and kayaking when used with a calibration adjustment.

**Supplementary Information:**

The online version contains supplementary material available at 10.1007/s40279-024-02131-z.

## Key Points

**Table:** 

Sports nutrition guidelines recommend carbohydrate intake be individualized to the athlete and modulated according to changes in training load, but there are limited methods to assess carbohydrate utilization during training sessions.
We examined bivariate relationships between both carbohydrate and energy expenditure during exercise and commonly used measures of training load, and then built prediction models to estimate carbohydrate and energy expenditure during exercise using training load and easily obtained measures of fitness.
We observed very large to near-perfect within-subject correlations between both carbohydrate and energy expenditure and all measures of training load. Prediction models displayed good accuracy in cycling, and have potential application in running or kayaking when used with a calibration adjustment.

## Introduction

Contemporary sports nutrition guidelines recommend carbohydrate intake be individualized to the athlete and their event, and modulated according to changes in exercise volume [1]. However, there are limited methods of assessing carbohydrate utilization during a given workout, leaving athletes and practitioners unclear as to how much carbohydrate or energy should be repleted. Indeed, there have been recent calls for a better understanding of the fuel costs and associated carbohydrate requirements of various training sessions commonly undertaken by athletes [2]. Based on the close relationship between mechanical work output and metabolic energy expenditure [3], it is plausible that readily available measures of exercise quantification (i.e., training load) could be used to model and predict carbohydrate utilization during exercise, particularly when combined with other measures obtained from traditional laboratory testing. However, historically there have been several challenges in studying this.

Carbohydrate utilization is estimated using indirect calorimetry, but this method is not valid during high-intensity intermittent exercise because of shifting acid–base balance and excess (non-oxidative) CO_2_ excretion through hyperpnea [4]. Changes in muscle glycogen are often used to estimate carbohydrate utilization, but this requires an invasive muscle biopsy with medical supervision and does not provide information on whole-body carbohydrate use. Furthermore, the level of muscle glycogen depletion is also specific at a subcellular and cellular level [5, 6], and therefore may vary with repeated sampling from the same individual, given the observed variability in muscle fiber-type distribution along a muscle’s length [7]. Another approach is to calculate the contribution of the three energy systems (aerobic, anaerobic alactic, and anaerobic lactic) during exercise based on measurements of oxygen uptake, the fast component of excess post-exercise oxygen uptake, and net changes in blood lactate level [8]. This method has been used across a range of sports including cycling [8], boxing [9], running [10], and rowing [11], resulting in an estimate of kilojoules (kJ) produced by each system. However, this does not consider the substrate (i.e., fat or carbohydrate) used for energy production or differences in efficiency with each substrate [12]. In combination, the traditional gas exchange measurement and the three-system approach to energy contributions could be used to estimate the total carbohydrate and energy cost of exercise at any intensity, but to our knowledge this has yet to be reported.

In the daily training environment, athletes and coaches routinely capture multiple training load indices, which can be measured and classified as either internal and/or external, based on the measurable aspects occurring internally or externally to the athlete [13]. Internal load reflects the relative physiological strain and disturbance in homeostasis of the metabolic processes in response to an external load, which is characterized by objective measures such as distance, power, or speed [14]. Because of the wide availability of cycling power meters, total work done during exercise (TWD) is a common measure of external training load for cyclists. Furthermore, very large correlations (*r* = 0.96–0.97) have been reported across multiple measures of internal and external training load in cyclists during racing and training [15], suggesting other metrics such as session rating of perceived exertion (sRPE) [16], Lucia training impulse (LuTRIMP) [17], and training stress score (TSS) [18] can also provide relevant information to athletes and coaches. However, there is no gold standard measure of training load [13], and measures of external and internal load are not always consistent. For example, TSS may overemphasize intensity compared with TWD, LuTRIMP, and sRPE [19, 20], but without a standard for comparison it is unclear which measure may be over-emphasizing or under-emphasizing intensity.

Given the strong theoretical and mechanistic links between measures of training load and both carbohydrate and total expenditure during exercise, it seems plausible that a strong association should exist between these phenomena, to the extent that practitioners may be able to estimate expenditure from training load metrics with reasonable precision. To our knowledge, this has yet to be explored. Therefore, to investigate the relationship between measures of training load and carbohydrate/energy expenditure, we used a novel method to estimate carbohydrate utilization from aerobic and anaerobic sources during moderate-intensity steady-state exercise and high-intensity interval training. The primary aims of our study were two-fold. The first was to quantify the bivariate relationships between both carbohydrate utilization and overall energy expenditure during exercise and commonly used measures of training load across sessions of varying duration and intensity. This answers the question “can training load be used as a proxy measure of carbohydrate/energy expenditure to quantify the correlation between an individual’s intake and exercise expenditure?”, as we have recently proposed [21]. The second aim was to model and predict carbohydrate and energy expenditure during exercise using measures of training load, alongside measures of cardiorespiratory fitness, dietary intake, and sex. This answers the question “can individuals estimate their carbohydrate/energy expenditure based on commonly available, non-invasive measures?”. In addition to using internal cross-validation of the prediction models, we also tested a separate set of athletes to quantify how well the model predictions would translate to a different set of athletes performing a different type of workout, across different exercise modalities (cycling, running, and kayaking). Finally, the study design also allowed us to examine day-to-day variability in heart rate (HR), rating of perceived exertion (RPE), carbohydrate oxidation, and oxygen consumption ($\dot{V}$O_2_) during low-intensity cycling.

## Methods

This study was undertaken in two parts: a primary study in cycling where participants performed four different laboratory-based training sessions, and a validation study where participants performed a single laboratory-based training session using one of three exercise modalities (cycling, running, or kayaking). Conceptually, this study includes a cross-sectional observational study (primary study) as well as a prediction model development and validation study (primary and validation studies). Accordingly, we adhered to the STROBE [22] and TRIPOD + AI [23] reporting guidelines where applicable.

### Participants

The primary study included 15 participants (ten male, five female), the validation study included 59 participants (41 male, 18 female). Sample size calculations are described in detail in Sect. 2.5. Participant characteristics are shown in Table 1. The study was open to all healthy male or female individuals aged 18–55 years regularly performing ≥ 3 h/week of training in the modality used for testing (cycle, run, or kayak). All interested participants that met the criteria were enrolled, and everyone enrolled completed all sessions. Study protocols and materials were approved by the Auckland University of Technology Ethics Committee (23/143 and 23/258).

**Table 1:** Participant characteristics

Characteristic	Cycle, primary *N* = 15^a^	Cycle, validation *N* = 21^a^	Kayak, validation *N* = 18^a^	Run, validation *N* = 20^a^
Sex				
Female	5 (33%)	7 (33%)	5 (28%)	6 (30%)
Male	10 (67%)	14 (67%)	13 (72%)	14 (70%)
Age (years)	34.2 (9.7)	31.6 (10.7)	29.7 (11.3)	28.0 (7.6)
Mass (kg)	74.8 (9.8)	73.7 (11.3)	76.7 (10.9)	70.0 (13.3)
BMI (kg/m^2^)	23.2 (1.8)	23.2 (2.3)	24.0 (2.0)	22.0 (2.2)
Training h/week	8.8 (3.1)	10.7 (6.6)	12.9 (4.2)	9.6 (5.1)
$\dot{V}$O_2max_ (mL/kg/min)	51.9 (7.2)	53.5 (11.0)	45.6 (4.8)	57.5 (7.2)
PPO (W)	355 (54)	341 (76)	164 (36)	351 (68)
VT_1_ (W)	167 (35)	175 (48)	78 (20)	249 (43)
VT_2_ (W)	246 (42)	240 (58)	109 (24)	289 (52)
Dietary CHO (g/kg)	3.7 (0.9)	4.1 (1.8)	3.6 (1.1)	4.5 (1.8)
Dietary fat (g/kg)	1.5 (0.4)	1.3 (0.5)	1.3 (0.3)	1.5 (0.8)
Dietary protein (g/kg)	1.8 (0.5)	1.6 (0.6)	1.8 (0.4)	2.1 (1.0)
Dietary energy (kcal/kg)	35.7 (7.6)	34.1 (11.6)	33.4 (6.6)	40.9 (14.2)

Dietary intake represents mean values from 4 days for each participant for the primary study and one day for each participant in the validation study

*BMI* body mass index, *CHO* carbohydrate, *PPO* peak power output, $\dot{V}$*O*_*2max*_ maximal oxygen consumption, *VT* ventilatory threshold

^a^*n* (%); mean (standard deviation)

### Primary Study

Participants reported to the laboratory on five occasions, with 1–7 days between sessions and without performing high-intensity sessions on consecutive days. Participants refrained from intense exercise and alcohol 24 h before each visit and avoided caffeine 16 h before each visit. Exercise was permitted the day before each session, with the duration and sRPE recorded using the Borg CR100® scale [24]. No exercise was allowed on the day of any laboratory visit. Participants were asked to maintain their normal dietary habits and recorded their intake for 1 day prior to each of visits 2–4 using a smartphone-based application that features foods from Australia and New Zealand (Easy Diet Diary, https://xyris.com.au/products/easy-diet-diary). All trials were conducted under standard laboratory conditions (18–20 °C, 40–65% relative humidity), with participants fan-cooled during exercise.

#### Visit 1

Participants reported to the laboratory in an overnight-fasted state. After obtaining written informed consent and completing a health screening, a graded exercise test was performed to determine ventilatory thresholds and maximal oxygen consumption ($\dot{V}$O_2max_). Participants cycled on an electronically braked cycle ergometer (Excalibur Sport; Lode BV, Groningen, The Netherlands), with expired gas collected and analyzed using a computerized metabolic system with mixing chamber (TrueOne2400; ParvoMedics, Sandy, UT, USA). The test began at 95 W, and power output increased by 35 W every 3 min until identification of the second ventilatory threshold (VT_2_), where the ventilatory equivalent for oxygen ($\dot{V}$E⋅$\dot{V}$O_2_^−1^) and carbon dioxide ($\dot{V}$E⋅$\dot{V}$CO_2_^−1^) increased alongside a reduction in PetCO_2_ [17]. Participants then cycled for 10 min at 100 W, followed by a step test starting at 150 W and increasing 30 W/min to task failure to obtain $\dot{V}$O_2max_. Thirty seconds following the test, a 0.3-μL blood sample was collected from the left index fingertip and analyzed immediately using a portable blood lactate analyzer (Lactate Pro 2, Carlton, VIC, Australia). The first ventilatory threshold (VT_1_) was identified as the work rate at which $\dot{V}$E⋅$\dot{V}$O_2_^−1^ began to increase in the absence of changes in $\dot{V}$E⋅$\dot{V}$CO_2_^−1^. Peak power (*W*_max_) was determined by the workload in the last completed stage plus the workload relative to the time spent in the last incomplete stage [power of completed stage + (30 × (seconds at uncompleted stage/60)], and $\dot{V}$O_2max_ and peak fat oxidation were recorded as the highest 15-s value from a moving average, calculated using the equation of Jeukendrup and Wallis [4] and a 1-s interpolation of breath-by-breath data.

#### Visits 2–5

In a randomized and counter-balanced order, participants completed four different training sessions as follows: (1) 90 min continuous cycling at 90% of VT_1_ power (low-intensity training [LIT] long), (2) 30 min continuous cycling at 90% of VT_1_ power (LIT-short), (3) 15 min continuous cycling at 90% of VT_1_ power, followed by two sets of 5 × 3-min intervals with 2 min recovery between intervals and 8 min recovery between sets (high-intensity interval training long, HIIT-long), and (4) 15 min continuous cycling at 90% of VT_1_ power, followed by two sets of 10 × 30-s intervals with 30-s recovery between intervals and 8-min recovery between sets (HIIT-short). All sessions were performed on the Lode cycle ergometer, with intervals performed using the cadence-dependent linear mode set to produce a workload of 110% VT_2_ power at their preferred cadence. Participants were instructed to produce their maximal power output across intervals. All recovery intervals were active recovery at 30% *W*_max_. All sessions were performed at the same time of morning (within 1 h) following an overnight fast. A standardized snack (Frooze balls, 27 g of CHO, 8 g of protein, 19 g of fat; Revive Foods, Auckland, New Zealand) was provided for participants to consume 45 min prior to starting exercise, and ad libitum water intake was allowed before and during the training session.

Music was played during all sessions except the graded exercise test. This was because many cyclists listen to music while training, particularly during extended duration indoor training sessions. Each participant selected their own playlist from a commercial streaming platform, which was repeated for each subsequent visit. Music was standardized based on individual preferences rather than playing the same music for all participants because of the influence of preferred versus non-preferred music on RPE during exercise [25]. An sRPE value was recorded 10–15 min following exercise using the Borg CR100^®^ scale, which offers additional precision compared with the CR10 scale [26]. Participants were familiarized with the CR100 scale in advance of the trials and given the scale for use at home 2 weeks prior to the first testing session.

**Fig. 1:**
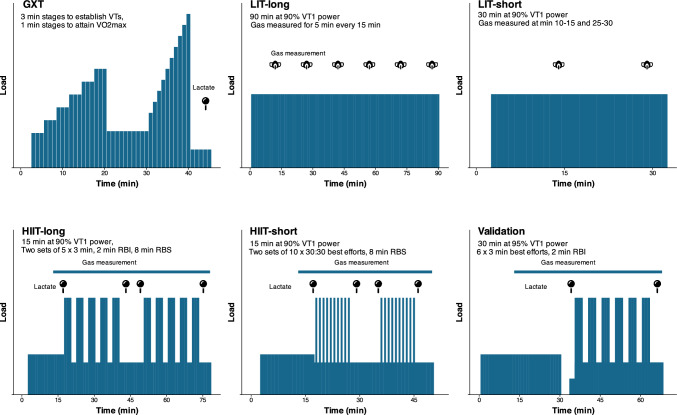
Schematic overview of the testing sessions. *GXT* graded exercise test, *HIIT* high-intensity interval training, *LIT* low-intensity training, *min* minutes, *RBI* rest between intervals, *RBS* recovery between sets, *VO2max* maximal oxygen consumption, *VTs* ventilatory thresholds, *VT1* first ventilatory threshold

Expired gas was measured during the last 6 min of every 15-min period during LIT sessions, and from minute 9 onwards during the HIIT sessions, with the exception of a 2-min break during minutes 6–7 of the recovery period between the two sets of intervals. Blood lactate level was measured 30 s before and 30 s after each interval set during the HIIT sessions. A schematic overview of the sessions for the primary and validation arms is shown in Fig. 1.

### Validation Study

To validate the prediction equations established in the primary study and to assess their generalizability to other exercise modalities, 59 additional participants were recruited to perform a graded exercise test and a single exercise session using either a cycling ergometer, motorized treadmill (h/p/cosmos, Nussdorf, Germany), or kayak ergometer (Dansprint, Hvidovre, Denmark). The graded exercise test was performed for cyclists as described above, whereas running tests began at 10 km/h and increased in 1-km/h increments, and kayaking tests began at 40 W (female participants) and 60 W (male participants) and increased in 15-W (female participants) and 20-W (male participants) increments. Participants returned to the laboratory 2–7 days later to perform a mixed-intensity exercise session consisting of 30 min of continuous exercise at 95% of VT_1_ power, followed by a 5-min recovery (5 min active recovery at 100 W for cyclists, or 3 min passive recovery followed by 2 min walking at 4 km/h for runners or 20 W on the kayak ergometer), and 6 × 3-min intervals with 2 min rest between intervals where participants were encouraged to give their best effort across the six intervals (Fig. 1). The cycling intervals were performed as described for the initial HIIT sessions, using the cadence-dependent linear mode. Treadmill intervals were set at 107% VT_2_ speed based on pilot testing. The intensity for kayak intervals was dictated by the participant. Recovery between intervals was set at 30% peak power output for cycling, 4 km/h for running, and 20% peak power output for kayak sessions.

The validation session was designed to be similar to a typical training session, but different from the sessions in the initial arm of the study. Session rating of perceived exertion was collected 10–15 min following exercise. Expired gas was measured during the last 6 minutesof every 15-min period during the 30-min continuous cycling, and for the remainder of the session. Lactate was measured 30 s before and 30 s after the interval set. A Stryd power meter (Stryd, Boulder, CO, USA) was used to collect running power data [27], and stroke-by-stroke power was collected from the kayak ergometer. To increase generalizability, validation sessions could be performed at any time of day, but subjects refrained from eating in the 4-h pre-exercise window, with the exception of the same standardized snack consumed 45 min prior to exercise.

### Data Analysis

Carbohydrate utilization and energy expenditure during exercise were determined separately for aerobic and anaerobic energy systems. For each exercise session, breath-by-breath gas exchange data were interpolated into second-by-second values using the *whippr* R package [28]. To determine the contribution from aerobic energy production, $\dot{V}$O_2_ values were converted to energy equivalents based on respiratory exchange ratio (RER) values using the conversion tables of Elia and Livesey [12]. This allows RER-specific energy conversions to be used. For example, the energy equivalent of 1 L of O_2_ is 4.687 kcal at an RER of 0.71, and 5.048 kcal at an RER of 1.0 [12]. This approach also allows the calculation of energy equivalents for RER values > 1.0 as CO_2_ is not needed for calculations [12]. To account for excess (non-oxidative) CO_2_ excretion, we considered 5.048 kcal/L as the maximum energy equivalent for the aerobic contribution if RER values were > 1.0. Energy equivalents were calculated on a second-by-second basis for the entire session (step 1). The RER value was then used to estimate the percentage of carbohydrate and fat oxidation using the conversions of Elia and Livesey [12]. The percentage carbohydrate contribution was multiplied by the energy equivalent to calculate energy from carbohydrate sources (step 2).

To convert from energy (kcal) to mass (g) of carbohydrate, consideration of exercise intensity is required. This is because the energy yield from carbohydrate varies depending on the source, with a range from 3.719 kcal/g of glucose to 4.187 kcal/g of glycogen [29]. The equations of Jeukendrup and Wallis [4] vary based on exercise intensity, assuming 50% of the carbohydrate oxidation is derived from plasma glucose and 50% from muscle glycogen during low-intensity exercise (40–50% $\dot{V}$O_2max_), and 20% from glucose and 80% from muscle glycogen at moderate-to-high intensity exercise (50–75% $\dot{V}$O_2max_). This results in carbohydrate oxidation yielding 3.95 kcal/g of carbohydrate during low-intensity exercise, and 4.07 kcal/g of carbohydrate during moderate-to-high intensity exercise [4]. It has also been recommended that resting analyses should assume 100% glucose oxidation [4]. With this in mind, we used a scaled approach whereby the percent contribution from glycogen was assumed to be equal to the exercise intensity as a percentage of $\dot{V}$O_2max_, allowing a second-by-second adjustment according to exercise intensity (step 3). The energy yield from glucose and glycogen-derived carbohydrate oxidation was then calculated and summed to get an intensity-adjusted energy yield from carbohydrate (step 4). The value for energy (kcal) from carbohydrate sources was divided by the adjusted energy yield to get a value of carbohydrate in grams for each second (step 5), with these values summed to yield a session total for grams of carbohydrate utilized through the aerobic energy pathways. A step-by-step example is shown in Box 1 for a $\dot{V}$O_2_ of 2.9 L/min and an RER of 0.93, for someone with a $\dot{V}$O_2max_ of 3.8 L/min.

**Box 1 Example Calculation of Aerobic Energy Production**

Step 1: Calculate energy expenditure per second.

RER of 0.93 yields 4.961 kcal/L O_2_/min

2.9 L × 4.961 kcal/L = 14.39 kcal/min/60 = 0.240 kcal/s

Step 2: Calculate energy expenditure from carbohydrate sources.

RER of 0.93 corresponds to a contribution from carbohydrate of 77.19%

0.240 kcal/s × 0.7719 = 0.185 kcal/s from carbohydrate

Step 3: Calculate percent contribution from glucose and glycogen sources, assuming percentage of glycogen is equivalent to percentage of $\dot{V}$O_2max_.

2.9 L/3.8 L = 76.3% from glycogen

1 − 76.3% = 23.7% from glucose sources

Step 4: Calculate energy yield from glucose-derived and glycogen-derived carbohydrate oxidation, summed for a total intensity-adjusted energy yield.

23.7% × 3.719 = 0.881 kcal/g from glucose

76.3% × 4.187 = 3.195 kcal/g from glycogen

0.881 + 3.195 = 4.076 kcal/g carbohydrate

Step 5: Calculate carbohydrate in grams per second.

0.185 kcal from carbohydrate per second (from step 2)/4.076 kcal/g (from step 4) = 0.045 g carbohydrate per second

Step 6: Calculate the sum of the second-by-second values to get a session total. Expired gas was recorded for the last 6 min of each 15-min block during low-intensity cycling, with the first minute of each collection period discarded. Therefore, values for the 5-min periods were multiplied by 3.

Energy produced from anaerobic lactate metabolism was determined using the net lactate accumulation, body mass, and O_2_ lactate equivalent method [3, 8], with example calculations shown in Box 2. Lactate was measured before and after the interval sets during the HIIT trials, with the change in lactate (post–pre) multiplied by 3 mL O_2_⋅kg^–1^⋅mmol⋅L^–1^ to create an oxygen equivalent [3], which was then multiplied by 21.1 kJ/L [8], and divided by 4.184 to convert from kJ to kcal (step 1).

To convert from energy (kcal) to mass (g) of carbohydrate, consideration of the ATP yields from anaerobic glycolysis and aerobic oxidation of carbohydrate is required. The net yield of anaerobic glycolysis is 2.9 ATP when starting from glycogen (assuming 90% α-1,4 glycosidic bonds) and 2 ATP when starting from glucose [30]. The complete oxidation of glycogen yields 34.35 ATP, and complete oxidation of glucose yields 33.45 ATP [30]. During high-intensity exercise, we assume the substrate for anaerobic glycolysis is glycogen, implying it would require 11.845 times more carbohydrate (because 34.35/2.9 = 11.845) to produce the same amount of ATP via anaerobic, compared with aerobic, metabolism. Based on the aerobic yield of 4.187 kcal/g of glycogen [12], we calculated grams of carbohydrate from anaerobic sources as kcal from step 1 divided by 4.187, multiplied by 11.845 to account for the inefficiency of ATP production from anaerobic glycolysis (step 2). This process was repeated for both interval sets. Total carbohydrate expenditure was calculated by summing the contributions from the aerobic and anaerobic systems. A step-by-step example of anaerobic energy calculation is shown in Box 2.

**Box 2. Example Calculation of Anaerobic Energy Production**

Step 1: Calculate kcal from anaerobic energy production.

Delta lactate = 9.3 mmol/L (post) − 1.5 mmol/L (pre) = 7.8 mmol/L

Oxygen equivalent = 3 × 7.8 × 70 kg body mass = 1638 mL O_2_ = 1.638 L O_2_.

1.638 L × 21.1 kJ/L = 34.56 kJ.

34.56 kJ/ 4.184 = 8.26 kcal via anaerobic energy production.

Step 2: Convert from kcal to grams of carbohydrate while accounting for the inefficiency of anaerobic energy production.

8.26 kcal/4.187 × 11.845 = 23.4 g carbohydrate

**Fig. 2:**
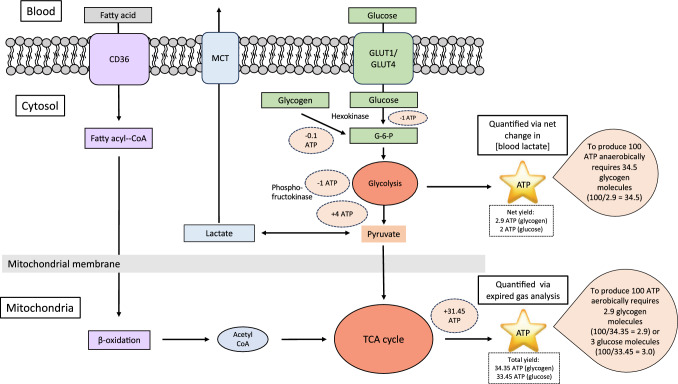
Overview of primary energy producing pathways in skeletal muscle. During glycolysis from glucose, 1 ATP is consumed at hexokinase and 1 ATP is consumed at phosphofructokinase to yield 2 trioses, each of which generates 1 ATP at phosphoglycerate kinase and 1 ATP at pyruvate kinase, for a net yield of 2 ATP/glucose. When starting from glycogen, less ATP is needed for the initial activation at hexokinase (~ 0.1 ATP), resulting in a greater net yield of 2.9 ATP [30]. An additional 31.45 ATP is produced from oxidative reactions, bringing the maximum total yield to 34.35 ATP from glycogen and 33.45 ATP from glucose. Glucose enters the cell via glucose transporters (GLUT)1 and 4. Lactate can be removed from the cell via monocarboxylate transporters (MCT). Fatty acids can enter the cell via fat transport proteins including cluster of differentiation 36 (CD36). Differences in efficiency are highlighted by a comparison of ATP production; to produce 100 ATP requires 34.5 glycogen molecules via anaerobic energy production or 2.9 glycogen molecules via aerobic energy production. *Acetyl CoA* acetyl coenzyme A, *TCA cycle* citric acid cycle

A visual overview of the pathways involved in energy production and rationale for this approach is provided in Fig. 2.

In addition to the strong theoretical and mechanistic rationale for this approach, we also tested our method using data from previously published studies that included metabolic tracers and/or muscle glycogen measurements. Calculations are provided in the Electronic Supplementary Material (ESM), showing good agreement (e.g., estimations of total carbohydrate utilization within ~ 1–4 g) when comparing our method with estimates using invasive techniques. For each exercise session, six measures of training load were calculated as shown in Table 2.

**Table 2:** Measures of training load and their calculations

Total work done	A measure of total mechanical energy spent (kJ), collected from the ergometer (cycling and kayaking) or Stryd power meter (running)
Session rating of perceived exertion-training load	Session rating of perceived exertion × duration (minutes) [16]. This value was divided by 10 to account for the 100-point scale, allowing easier comparisons to other research using the 10-point scale
LuTRIMP	(Duration (minutes) in zone 1 × 1) + (duration in zone 2 × 2) + (duration in zone 3 × 3). Zones were calculated using power (LuTRIMP power) and HR (LuTRIMP HR), with zone 1 below VT_1_, zone 2 between VT_1_ and VT_2_, and zone 3 above VT_2_ [17]
TSS	[(seconds × NP × IF)/(FTP × 3600)] × 100 [18]. Power at VT_2_ was used as an estimate of FTP, as has been done by others [31]. NP is calculated by creating rolling 30-s averages and raising each value to the fourth power, then taking the fourth root of the average of the fourth powers. IF is calculated as NP/FTP
HR TSS	Calculated the same as TSS, substituting HR for power and using VT_2_ HR as a measure of functional threshold HR

*FTP* functional threshold power, *HR* heart rate, *IF* intensity factor, *LuTRIMP* Lucia training impulse, *NP* normalized power, *TSS* training stress score, *VT*_*1*_, first entilator threshold, *VT*_*2*_ second entilator threshold

### Statistical Analysis

#### Primary Study

To estimate differences in training load across the four sessions for each metric, a series of linear mixed models were fitted using the *lme4* R package with training load as the dependent variable, session as a fixed factor, and participant ID as a random intercept. Model-estimated means were calculated using the *emmeans* R package and contrasts between each session (within each training load metric) were estimated using the Holm correction for multiple comparisons. To examine the bivariate relationship between training load measures and the total carbohydrate and energy cost of exercise, a repeated-measures correlation was performed using the *rmcorr* R package, which allows analysis of repeated-measures data without violating independence assumptions [32]. To examine day-to-day variation, all four trials began with the same 15-min period of cycling at 90% VT_1_ power, allowing us to compute the typical error of measurement for *V*O_2_, HR, carbohydrate oxidation, and RPE according to the approach of Hopkins [33].

To predict carbohydrate utilization and energy expenditure based on training load and other commonly measured variables known to influence substrate selection such as $\dot{V}$O_2max_, sex, and dietary intake [34], multivariable models were created for each of the six training load measures predicting each of the two dependent variables (energy expenditure and carbohydrate utilization) using generalized estimating equations. Generalized estimating equation models provide population-averaged (e.g., marginal), rather than subject-specific models while accounting for repeated measurements within participants [35]. The Quasi Information Criterion was used for selecting an independence correlation structure as the working correlation matrix [36]. The following variables were considered for the full models: training load, training load^2^, session duration (minutes), session duration^2^, prior-day sRPE training load (sRPE-TL), type of session (continuous or interval training), prior day dietary carbohydrate and fat intake (g/kg), $\dot{V}$O_2_ at VT_2_ (L/min and % $\dot{V}$O_2max_), $\dot{V}$O_2max_ (mL/kg/min and L/min), blood lactate at the end of the $\dot{V}$O_2max_ test, peak fat oxidation (g/min), and sex. The following pre-specified interactions were also considered in the full model: prior-day sRPE-TL × prior day carbohydrate intake, session duration × training load, type of session × training load, type of session × $\dot{V}$O_2max_, and type of session × $\dot{V}$O_2_ at VT_2_.

The top candidate models were identified using the *glmulti* R package [37], which performs a genetic search across possible models specified by a given set of predictors and selects the top models according to the corrected Akaike Information Criterion. From the reduced pool of models, we performed participant-level leave-one-out cross-validation, which fits a series of models on all but one of the participants, whose four sessions are used as a hold-out testing set [38], selecting the model with the lowest mean absolute error (MAE) as the final model for each measure of training load. The fit of each model was checked by visualizing the Q–Q and other residual plots to ensure approximate residual normality and homoscedasticity using the *performance* R package. Model performance is reported as the coefficient of determination (*R*^2^), which represents the proportion of variance explained by the model, and the MAE, which quantifies the average absolute discrepancy between the observed and predicted values. These metrics were calculated using both in-sample data (i.e., the same data used to train the model and evaluate performance) and cross-validation, which offers a more realistic and unbiased (or least biased) estimate of model performance in the population in which the model is intended [39]. Performance metrics for cross-validation are reported as mean [95% confidence intervals]. There were no missing data for models in the primary study.

#### Validation Study

Data from the validation sessions were analyzed in the same manner as the primary study, with each session analyzed as only the 30-min low-intensity portion, and as the full session (30-min low-intensity and high-intensity intervals). Values of total carbohydrate utilization and energy expenditure for each session were predicted from the previously fit models for each measure of training load. Model performance was assessed using measures of overall fit *R*^2^ (proportion of variance in explained in the external validation dataset, calculated using the traditional definition with sum of squares rather than the correlation between predicted and actual values) and MAE, and assessed for calibration, which was quantified as calibration-in-the-large (the difference between mean observed and mean predicted outcome values, with 0 being ideal) and calibration slope (the agreement between predicted and observed values across the range of predicted values, with a slope of 1 being ideal) [39]. Finally, models were recalibrated using the intercept and slope of a linear model regressing the actual values on the predicted values [40], with measures of *R*^2^ and MAE reported on the calibrated data. Because of technical issues, data for the low-intensity portion of one kayak trial and the total work done for one running trial were missing. Rather than using imputation, these data points were omitted from the predictions. One other kayaking trial consisted of only three intervals because of an equipment malfunction but data from the first 45 min of the session were included for analysis.

Based on Riley et al. [41], a minimum sample size of 15 was calculated for the primary study. This calculation used an estimated adjusted *R*^2^ value of 0.89 and considered a model with up to six predictors. The choice of six predictors was derived from Riley et al. [42], to ensure a shrinkage factor of at least 0.9 and to maintain a difference between adjusted and apparent *R*^2^ values below 0.05. However, our sample size is below the minimum size of 240 (using the rule of 234 + number of predictors) needed for precise estimates of the residual standard deviation [41]. This means there will still be some uncertainty in the parameter estimates that can only be solved with very large sample sizes that extend beyond the capacity of this project. The approach of Archer et al. [43] was used to calculate the minimum sample sizes needed in the validation dataset to obtain precise estimates of *R*^2^, calibration-in-the-large, and calibration slope, assuming 90% confidence intervals with target widths of 0.2 for *R*^2^ and 0.2 for the calibration slope. Calculations were made separately for each model, resulting in a minimum requirement of 7–11 participants (kcal) and 13–19 participants (carbohydrate) in each validation arm depending on the model (Table 1 and R code in the ESM). For the kayaking arm of the validation study, we were only able to recruit 18 athletes, which is sufficient for all energy expenditure models and five of the six carbohydrate models, but just below the target sample size of 19 for sRPE-TL.

To determine the minimum sample size for detecting differences in training load across the four sessions in the primary study, we calculated the means and standard deviations for each session, estimated the pooled standard deviation, and determined the effect size (Cohen’s *f*). Using these values, we performed a power analysis that indicated that a sample size of six participants was required to achieve 95% power at a 5% significance level. All analyses were carried out with R version 4.3.1 (The R Foundation for Statistical Computing, Vienna, Austria). Descriptive statistics are provided as mean ± standard deviation, statistical significance was accepted at *p* < 0.05.

## Results

**Fig. 3:**
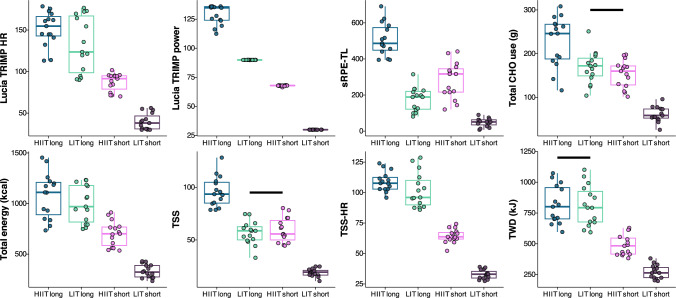
Training load and energy and carbohydrate (CHO) use for each training session in the primary study. *Solid black lines* indicate no significant difference between sessions (*p* > 0.05). *HIIT* high-intensity interval training, *HR* heart rate, *LIT* low-intensity training, *sRPE-TL* session rating of perceived exertion training load, *TRIMP* training impulse, *TSS* training stress score, *TSS-HR* TSS calculated with heart rate, *TWD-kJ* total work done (kJ)

**Fig. 4:**
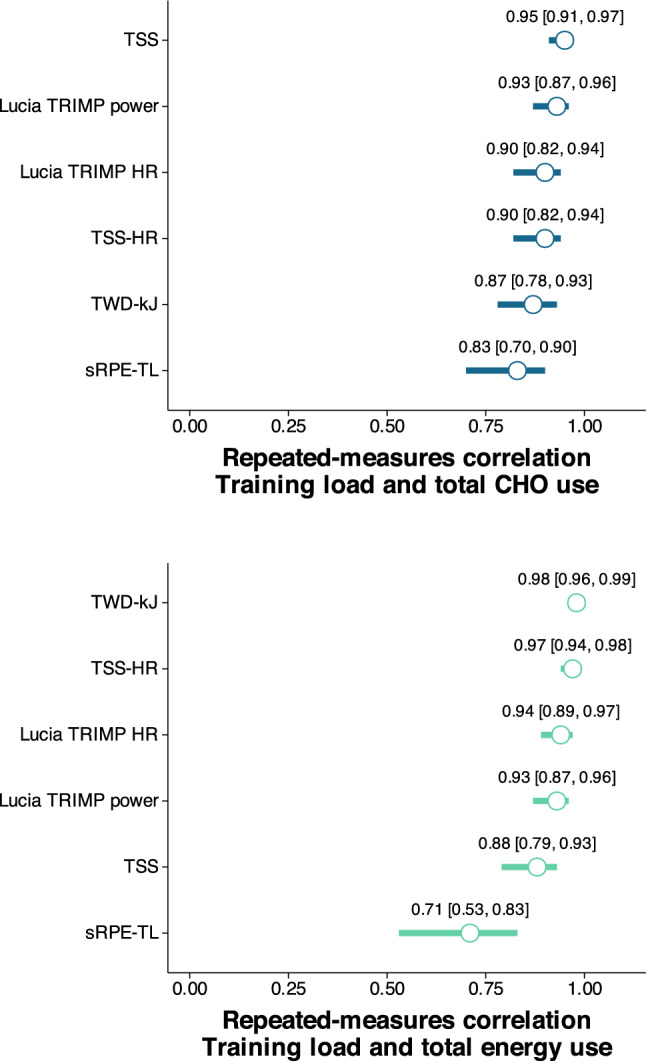
Repeated-measures correlation values with 95% confidence intervals between training load measures and carbohydrate (CHO, *top*) and energy use (*bottom*) from the primary study. *HR* heart rate, *sRPE-TL* session rating of perceived exertion training load, *TRIMP* training impulse, *TSS* training stress score, *TSS-HR* TSS calculated with heart rate, *TWD-kJ* total work done (kJ)

Participant characteristics and self-selected dietary intake are shown in Table 1. The typical error of measurement of day-to-day variation during low-intensity cycling was 2.9 beats/min for HR, 0.08 L/min for $\dot{V}$O_2_, 0.21 g/min for carbohydrate oxidation, and 4.0 arbitrary units (0–100 AU scale) for RPE. Training load, energy expenditure, and carbohydrate utilization for each training session are shown in Fig. 3. All measures of training load were significantly different for each training session, except total carbohydrate utilization and TSS, which were not different between LIT-long and HIIT-short, and TWD-kJ, which was not different between HIIT-long and LIT-long. A repeated-measures correlation analysis indicated very large to near-perfect correlations (0.71–0.98) between all training load metrics and both outcomes (Fig. 4).

Multivariable regression models were created for each of the six training load measures predicting the two dependent variables (energy expenditure and carbohydrate utilization, resulting in 12 unique models), with additional variables that can be easily obtained from routine exercise physiology testing. The best models were selected using cross-validation, resulting in a unique set of predictors for each model. Model coefficients for the included variables are shown in Table 3, along with model performance metrics *R*^2^ and MAE using both in-sample data and leave-one-participant out cross-validation. All models explained a large amount of variance (*R*^2^ values of 0.77–0.96) and displayed good accuracy (MAE of 16–21 g [10–14%] of carbohydrate and 53–82 kcal [7–11%]).

**Table 3:** Model performance measures and coefficients

	*R*^2^	cv-R^2^	MAE	cv-MAE	Intercept	Type, continuous	Sex, female	TL	TL^2^	Duration (min)	VT2 VO_2_ (L)	VT2 (%max)	*V*O_2max_ (L)	*V*O_2max_ (mL/kg/min)	Prior-day sRPE-TL
CHO															
TSS-HR	0.93	0.84 [0.74, 0.94]	16.0	17.6 [14.4, 20.8]	− 22.77 (18.23)	− 42.77 (5.85)	− 28.83 (6.43)	1.585 (0.086)					23.62 (3.65)		− 0.039 (0.007)
TSS	0.92	0.82 [0.71, 0.93]	16.1	18.2 [14.3, 22.1]	− 28.01 (23.51)	− 38.39 (7)	− 21.69 (7.48)		0.007 (0.001)	1.43 (0.12)	32.51 (6.51)				− 0.062 (0.012)
TWD-kJ	0.92	0.85 [0.77, 0.93]	16.5	17.9 [13.8, 22]	76.52 (9.3)	− 49.93 (6.26)	− 22.37 (4.92)	0.201 (0.009)							− 0.06 (0.011)
Lucia TRIMP power	0.89	0.77 [0.62, 0.91]	18.2	20.1 [15.8, 24.4]	− 69.16 (16.76)	− 50.39 (7.23)			0.003 (0.001)	1.39 (0.14)	46.83 (4.56)				− 0.058 (0.015)
Lucia TRIMP HR	0.90	0.79 [0.68, 0.89]	18.4	21.2 [17.6, 24.9]	− 21.36 (31.21)	− 61.47 (7.22)	− 16.26 (9.38)		0.002 (0.001)	1.4 (0.2)	35.24 (8.85)				− 0.046 (0.009)
sRPE-TL	0.88	0.77 [0.66, 0.88]	19.1	21.2 [16.8, 25.6]	− 75.89 (16.46)	− 54.53 (8.67)			1e−04 (4e−05)	1.8 (0.13)	46.45 (4.75)				− 0.058 (0.014)
Kcal															
TWD-kJ	0.96	0.93 [0.89, 0.97]	52.6	58.5 [38, 78.9]	135.04 (34.97)	− 95.68 (15.56)	− 41.79 (23.86)	1.18 (0.036)							
TSS	0.96	0.92 [0.89, 0.96]	55.1	59.6 [48, 71.1]	− 709.07 (42.34)			5.3 (0.288)		7.56 (0.43)	225.35 (13.96)				
TSS-HR	0.94	0.90 [0.85, 0.95]	61.7	72.2 [54.3, 90.1]	− 772.78 (198.04)	− 116.84 (23.67)	− 37.65 (24.65)	5.12 (0.807)		5.23 (1.2)		3.23 (1.615)	164.73 (21.65)		
Lucia TRIMP HR	0.93	0.88 [0.82, 0.93]	69.1	79.4 [62.3, 96.6]	− 830.95 (261.22)	− 177.24 (25.05)			0.006 (0.002)	9.7 (0.77)		4.376 (2.359)	172.96 (19.11)		
Lucia TRIMP power	0.93	0.88 [0.83, 0.93]	72.0	78.6 [63.6, 93.7]	− 786.48 (71.93)			10.5 (1.619)	− 0.03 (0.007)	4.05 (1.07)	222.81 (20.51)				
sRPE-TL	0.93	0.88 [0.83, 0.92]	73.1	81.7 [63.8, 99.6]	− 615.45 (60.89)	− 132.4 (40.94)		0.2576 (0.138)		10.69 (0.68)	208.62 (29.05)			1.453 (1.163)	

Model coefficients are shown with (standard error)

*CHO* carbohydrate, *cv-MAE* MAE from cross-validation, *cv-R*^*2*^ R^2^ from cross-validation, *MAE* mean absolute error, *min* minutes, *TL* training load, *TL*^*2*^ training load^2^, *VT2* (%max) is on 0–100 scale, *%max* percentage maximum

**Fig. 5:**
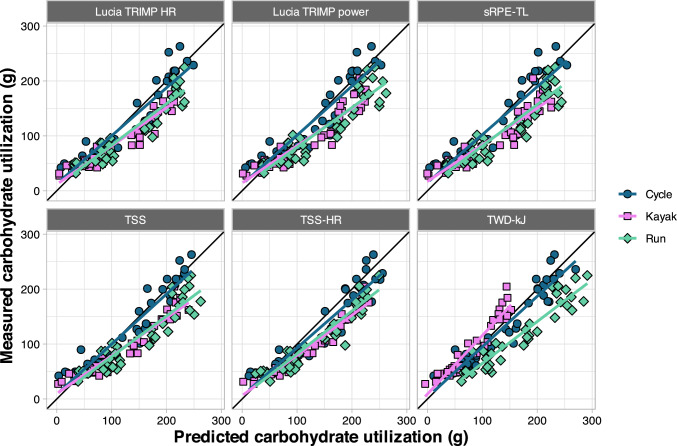
Predicted versus measured values of carbohydrate utilization during validation sessions, separated by exercise mode. *HR* heart rate, *sRPE-TL* session rating of perceived exertion training load, *TRIMP* training impulse, *TSS* training stress score using power, *TSS-HR* training stress score calculated using HR, *TWD-kJ* total work done (kJ)

**Fig. 6:**
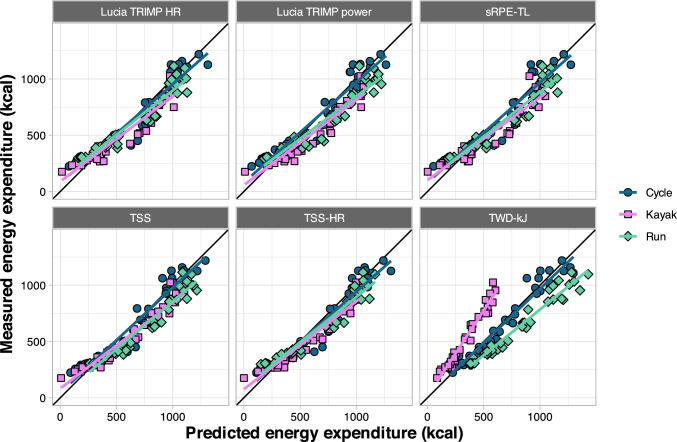
Predicted versus measured values of energy expenditure during validation sessions, separated by exercise mode. *HR* heart rate, *sRPE-TL* session rating of perceived exertion training load, *TRIMP* training impulse, *TSS* training stress score using power, *TSS-HR* training stress score calculated using HR, *TWD-kJ* total work done (kJ)

To test the application and generalizability of the models, we performed a validation study using a different training session, and three new groups of athletes (cyclists, runners, and kayakers). Model-predicted values are shown compared to actual values for carbohydrate utilization (Fig. 5) and energy expenditure (Fig. 6). Summary values shown in Table 4, including MAE and *R*^2^ (calculated using observed and model-predicted values), calibration-in-the-large (difference between the mean observed and the mean predicted outcome values), calibration intercept, and slope (from regressing the actual values on the predicted values in a linear model), and MAE and *R*^2^ (calculated using the calibration-adjusted predictions and the actual values).

**Table 4:** Model accuracy from the validation study

	MAE, raw	*R*^2^, raw	CITL	Calibration intercept	Calibration slope	MAE, calibrated	R^2^, calibrated
Carbohydrate utilization (g), cycle							
sRPE-TL	21.9	0.85	− 0.8	14.9	0.87	20.3	0.87
TSS	18.3	0.90	− 2.3	8.7	0.91	17.8	0.91
TSS-HR	23.5	0.84	− 11.2	5.8	0.87	19.3	0.89
TWD-kJ	16.7	0.90	− 7.4	3.3	0.92	15.0	0.92
Lucia TRIMP HR	20.5	0.86	− 3.2	12.7	0.87	19.1	0.88
Lucia TRIMP power	20.7	0.87	− 1.5	11.7	0.89	19.6	0.88
Carbohydrate utilization (g), kayak							
sRPE-TL	29.9	0.50	− 20.2	17.0	0.67	15.0	0.87
TSS	31.9	0.45	− 27.2	9.8	0.69	11.2	0.92
TSS-HR	28.9	0.58	− 25.2	6.1	0.73	8.5	0.95
TWD-kJ	16.1	0.84	14.2	9.0	1.07	11.7	0.92
Lucia TRIMP HR	28.9	0.53	− 23.0	11.7	0.70	13.0	0.89
Lucia TRIMP power	30.1	0.49	− 22.7	14.1	0.68	14.3	0.88
Carbohydrate utilization (g), run							
sRPE-TL	32.0	0.52	− 28.4	6.3	0.75	18.6	0.86
TSS	36.9	0.38	− 35.4	2.7	0.74	18.4	0.86
TSS-HR	29.5	0.62	− 25.5	4.4	0.78	15.0	0.90
TWD-kJ	50.4	0.08	− 50.0	-11.1	0.76	15.7	0.90
Lucia TRIMP HR	29.7	0.58	− 25.6	7.5	0.76	16.4	0.88
Lucia TRIMP power	36.4	0.40	− 33.7	6.3	0.73	18.5	0.86
Energy expenditure (kcal), cycle							
sRPE-TL	79.9	0.90	8.7	80.2	0.89	70.6	0.92
TSS	68.1	0.93	2.2	45.0	0.93	65.3	0.94
TSS-HR	80.3	0.91	− 20.7	51.9	0.89	72.4	0.92
TWD-kJ	53.0	0.95	− 18.5	9.4	0.96	48.3	0.96
Lucia TRIMP HR	75.7	0.91	− 3.0	73.9	0.88	69.8	0.92
Lucia TRIMP power	78.0	0.91	11.1	79.7	0.89	68.6	0.93
Energy expenditure (kcal), kayak							
sRPE-TL	106.3	0.76	− 43.1	100.8	0.74	59.7	0.90
TSS	91.1	0.82	− 46.7	82.7	0.76	45.1	0.95
TSS-HR	81.9	0.84	− 43.5	75.5	0.78	43.4	0.95
TWD-kJ	181.8	0.32	181.8	14.3	1.52	36.0	0.97
Lucia TRIMP HR	96.8	0.79	− 43.8	88.8	0.76	59.5	0.91
Lucia TRIMP power	114.0	0.70	− 86.2	56.0	0.76	58.6	0.92
Energy expenditure (kcal), run							
sRPE-TL	91.6	0.83	− 48.5	93.6	0.79	58.4	0.92
TSS	113.0	0.76	− 103.2	11.1	0.84	54.1	0.94
TSS-HR	81.4	0.86	− 38.1	90.2	0.81	55.1	0.94
TWD-kJ	178.3	0.47	− 178.3	− 28.1	0.81	39.5	0.96
Lucia TRIMP HR	85.2	0.85	− 28.9	107.9	0.79	58.0	0.92
Lucia TRIMP power	125.9	0.71	− 82.6	94.0	0.75	65.9	0.91

*CITL* calibration-in-the-large (difference between the mean observed and the mean predicted outcome values), *MAE* mean absolute error, *MAE-raw* MAE of predicted values, MAE-calibrated MAE for each model following adjustment of each prediction as the calibration intercept + (predicted value × calibration slope), *sRPE-TL* session rating of perceived exertion training load, *TSS* training stress score calculated using power, *TSS-HR* training stress score calculated using HR, *TWD-kJ* total work done (kJ)

*R*^2^ values represent the coefficient of determination calculated using the traditional definition with sum of squares rather than the correlation between predicted and actual values

The predictions for cycling displayed the highest accuracy, and most predictions were higher than actual values apart from TWD-kJ for kayak. This is evidenced by negative values for calibration-in-the-large and calibration slope values less than 1 (Table 4). The MAE ranged from 16.1 to 50.4 g [15–45%] for carbohydrate utilization models and 53.0–181.8 kcal [9–31%] for energy expenditure models.

**Fig. 7:**
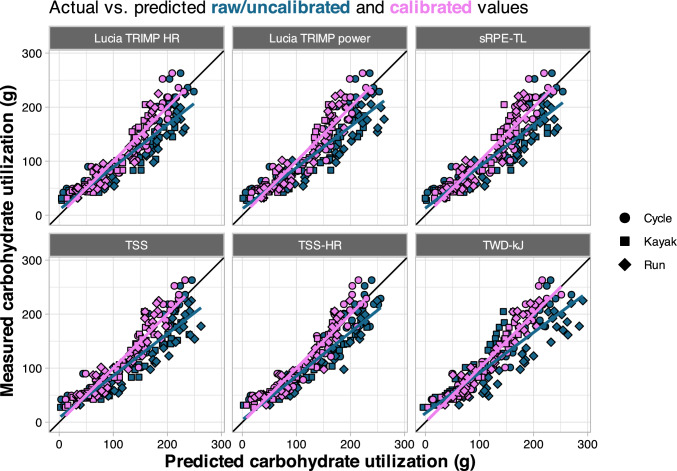
Predicted versus measured values for calibrated carbohydrate models, with shapes denoting each sport and colors depicting the raw/uncalibrated values (*blue*) and calibrated values (*violet*). *HR* heart rate, *sRPE-TL* session rating of perceived exertion training load, *TRIMP* training impulse, *TSS* training stress score using power, *TSS-HR* training stress score calculated using HR, *TWD-kJ* total work done (kJ)

**Fig. 8:**
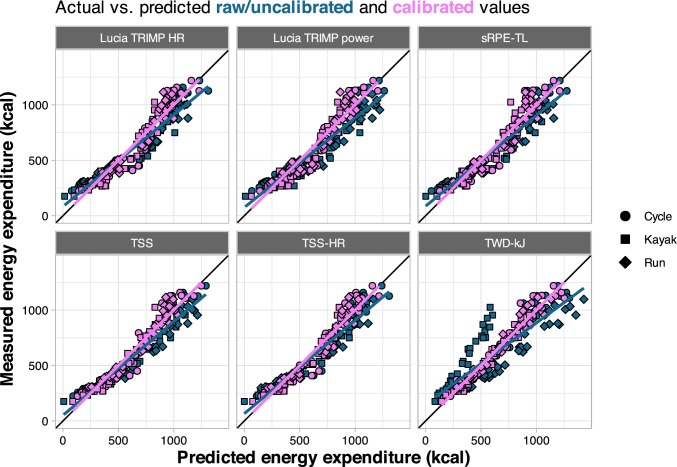
Predicted versus measured values for calibrated energy expenditure models, with shapes denoting each sport and colors depicting the raw/uncalibrated values (*blue*) and calibrated values (*violet*). *HR* heart rate, *sRPE-TL* session rating of perceived exertion training load, *TRIMP* training impulse, *TSS* training stress score using power, *TSS-HR* training stress score calculated using HR, *TWD-kJ* total work done (kJ)

After applying the calibration adjustment to each predicted value in the validation study (calibration intercept + predicted value × calibration slope), accuracy of all models was improved as shown in Fig. 7 (carbohydrate utilization) and Fig. 8 (energy expenditure). The calibrated MAE ranged from 8.5–20.3 g [8–18%] for carbohydrate utilization models to 36.0–72.4 kcal [6–12%] for energy expenditure models (Table 4).

## Discussion

The main findings of our study were: (1) common measures of training load display very large to near-perfect associations (*r* = 0.71–0.98) with both carbohydrate utilization and energy expenditure during exercise, (2) TSS was the only measure of training load to accurately reflect similar total carbohydrate utilization between the longer duration low-intensity session and the shorter duration high-intensity session, (3) carbohydrate utilization and energy expenditure during cycling could be predicted with a high degree of accuracy (MAE 17–24 g of carbohydrate, 53–80 kcal) using measures of training load along with easily obtainable laboratory measures, and (4) these models can be applied in running and kayaking when used with a calibration adjustment.

A key aim of the study was to quantify the bivariate relationships between both carbohydrate utilization and overall energy expenditure during exercise and commonly used measures of training load across sessions of varying duration and intensity. This investigated the question “can training load be used as a proxy measure of carbohydrate/energy expenditure to quantify the correlation between an athlete’s intake and exercise expenditure?”, as we have recently proposed [21]. Repeated-measures correlations showed very large to near-perfect relationships between each of the training load measures and both carbohydrate utilization and energy expenditure, supporting the use of any of the training load measures by athletes looking for a way to compare their carbohydrate or energy intake with their exercise. The weakest relationships were observed for sRPE-TL, but the correlation values of 0.71–0.83 would still be considered very large and sRPE-TL is a more pragmatic option for many athletes and practitioners.

Despite the ubiquity of training load quantification and variety of methods for measuring load, there is no consensus on which methods best represent the true load of a training session [13, 31, 44]. This is context dependent and relies heavily on the nature of the exercise stimulus (e.g., the sport or mode of training). Some suggest TSS is influenced more by intensity than other training load metrics such as TWD, LuTRIMP, and sRPE-TL, meaning TSS will be different for sessions where the same amount of energy is expended either at low or high intensity [19, 20]. This may be related to the quadratic effect of exercise intensity on normalized power (a component of the TSS calculation), whereas most other measures of training load feature linear or exponential relationships [20]. The rationale for this quadratic relationship has been questioned based on the lack of quadratic relationship between exercise intensity and measures of internal load such as RPE, $\dot{V}$O_2_, HR, blood lactate, as well as biochemical and hormonal responses [20]. However, the close relationship we observed between TSS and total carbohydrate use during exercise (Fig. 4), along with the observation that only TSS could accurately differentiate the carbohydrate needs of the various sessions (Fig. 3), suggests the quadratic relationship between TSS and exercise intensity may be a “feature”, rather than a “bug”. The inefficiency of the anaerobic energy pathways [30] offers a mechanism for why TSS might most accurately reflect total carbohydrate use during exercise of varying intensities. There has also been concern of a misestimation of the physiological impact of a long easy endurance ride compared with a short high-intensity ride when expending the same amount of energy [20]. This concern is reflected in our study by the higher values of sRPE-TL for the HIIT-short compared with LIT-long sessions, despite TWD being higher for the LIT-long session, and TSS being the same between the two sessions (Fig. 3).

It has been suggested that studies comparing different training intensities should equalize sessions in terms of energy expenditure or work [45]. However, work accumulated in high-intensity zones impacts performance [46] and training adaptations [47] differently than work-matched exercise performed in lower intensity zones. Based on our data, TSS could be considered as a viable alternative for matching training load between groups, and/or could be used to adjust for differences between high-intensity and low-intensity training groups. When combined with the known influence of post-exercise muscle glycogen levels on the molecular adaptations to exercise [48, 49], our findings can help explain the advantage of high-intensity training, compared with moderate-intensity training, commonly observed when total work is matched between groups [47, 50–52]. Retrospective analyses of training studies comparing moderate-intensity and high-intensity training using TSS could further explore this hypothesis.

This study used a novel method of estimating carbohydrate utilization and energy expenditure during exercise, extending methodological approaches from adjacent fields of biochemistry, physiology, and nutrition. Several challenges had to be overcome including energy conversions that are dependent on the relative contributions of fat and carbohydrate, the contributions of glucose and glycogen, differences in aerobic and anaerobic metabolism, the non-validity of gas exchange measures during high-intensity exercise, and the computational challenges associated with large data sets.

Carbohydrate oxidation is often calculated using measures of $\dot{V}$O_2_ and $\dot{V}$CO_2_, but this method assumes a stable bicarbonate pool and is thus unreliable at intensities above ~ 75% $\dot{V}$O_2max_ because of a shifting acid–base balance and excess (non-oxidative) CO_2_ being excreted through hyperpnea [4]. Our approach, using the $\dot{V}$O_2_-based calculations of Elia and Livesey [12], allows aerobic energy expenditure to be calculated during higher intensities, and a comparison of the two calculation methods during low-intensity exercise revealed near-perfect correlation values of 0.97–0.99 (data not shown). Although lactate is produced during steady-state exercise, a major portion is eliminated through oxidation and so the rate of oxygen consumption effectively accounts for and reflects the energy generated via aerobic glycolysis [53].

Data were analyzed on a second-by-second basis, which allowed continuous adjustment of the energy yield of O_2_ based on substrate utilization, and the intensity-dependent changes in the relative contribution of glucose and glycogen for carbohydrate oxidation. For the latter, we chose a scaled approach whereby the percent contribution from glycogen was assumed to be equal to the exercise intensity as a percentage of $\dot{V}$O_2max_. This assumption was made based on the recommendations of Jeukendrup and Wallis [4] that resting analyses should assume 100% glucose oxidation, exercise around 40–50% $\dot{V}$O_2max_ can assume 50% of carbohydrate oxidation is derived from plasma glucose and 50% from muscle glycogen, and exercise up to ~ 75% $\dot{V}$O_2max_ can assume 20% from glucose and 80% from muscle glycogen. A limitation of this approach is that the estimations assume normal glycogen levels, yet the balance between glucose and glycogen-derived energy production is likely to change as the exercise duration extends and glycogen is depleted [54, 55]. However, the practical difference this would make to the carbohydrate calculation would be quite small as the substrate shifts more towards plasma glucose and away from muscle glycogen. We also adjusted the energy yield of O_2_ based on substrate utilization. This is in contrast with many prior studies using a single value (e.g., 20.9 kJ/L O_2_), an approach that has been criticized [56].

To account for differences in efficiency of anaerobic and aerobic energy production, a conversion factor was used based on work showing the complete oxidation of glycogen yields 34.35 ATP and the net yield of anaerobic glycolysis is 2.9 ATP [30]. Assuming the primary substrate during high-intensity exercise is muscle glycogen, this implies 11.845 times more carbohydrate would be required to produce the same amount of ATP via anaerobic, compared with aerobic, metabolism (Fig. 2). This inefficiency explains the observations of extremely high rates of muscle glycogen breakdown (~ 85–111 mmol/kg/dry mass) following a single 30-s bout of maximal cycling [57, 58]. For comparison, 120 min of cycling at 65–75% *V*O_2max_ would result in a similar depletion of muscle glycogen [59, 60]. Furthermore, our estimated values for energy contribution from anaerobic metabolism are in line with the previously reported range of 15–20 kcal non-oxidative energy capacity for a 70-kg human [53, data not shown]. Although this approach would be challenging to validate with any single method, it is derived from well-established methods for energy system quantification and displays very close agreement with two different approaches to quantification carbohydrate utilization, described in detail in the ESM. Collectively, our approach overcomes the methodological challenges that have previously precluded this type of analysis from being performed. We also believe this approach can be extended to other exercise modalities and to different types of training sessions.

The second aim of the study was to model and predict carbohydrate utilization and energy expenditure during exercise using measures of training load. Models were built in the primary study of cyclists performing four laboratory-based training sessions. The best model for each training load metric was selected from a pool of models containing commonly used and easily obtainable measures such as $\dot{V}$O_2max_, ventilatory thresholds, dietary intake, sex, and prior-day training load. Using internal validation (i.e., testing the model on the same data that were used to train the model), *R*^2^ values ranged from 0.88 to 0.96 and MAE values ranged from 16.0 to 19.1 g for carbohydrate utilization and from 52.7 to 73.2 kcal for energy expenditure. This is referred to as ‘apparent performance’, which, although commonly the only approach used in the field of sports science, typically provides overly optimistic values compared with when the model is evaluated in new data [39]. Therefore, it is recommended that prediction models should have additional internal–external, and external validation [61]. Internal–external validation refers to using a portion of the data for training a model and a separate portion of data for testing it. Because each participant in the primary study performed four training sessions, we used k-fold cross-validation splitting the data by participant. This means each participant was left out once for assessment of a model fit on all other participants, with the reported metrics based on the pooled assessment data [61]. Using this cross-validation approach in the primary study, *R*^2^ values ranged from 0.77 to 0.93 and MAE values ranged from 17.6 to 21.2 g for carbohydrate utilization and from 58.5 to 81.7 kcal for energy expenditure, representing a reasonable and expected decline in performance from the apparent performance (Table 3). From a practical perspective, the model prediction errors were lower than the errors from dietary quantification performed by sports nutritionists, which have been reported to be ~ 20–65 g of carbohydrate and ~ 140–369 kcal/day [62].

External validation was then used to quantify how well the model predictions translated to a different set of athletes performing a different type of workout, across different exercise modalities. To this end, we recruited 59 additional athletes to perform a single in-laboratory training session using one of three exercise modalities (cycling, running, and kayaking). Overall, predictions for cycling were better than running and kayaking, and the between-sport differences were highly consistent (Figs. 5 and 6). This implies differences inherent to performing the sport and/or the measurement devices used for each sport are responsible for much of the prediction error, rather than simply individual variation and/or noise in the data. This idea is further supported by the pronounced improvements with model calibration (Table 4, Figs. 7 and 8), which adjusts the predictions using a simple linear model. For example, the greatest overpredictions were the run models that used TWD-kJ (shown as the most negative calibration-in-the-large values in Table 4). Although it is acknowledged that estimating mechanical power for running is considerably more complex than it is for cycling, we chose to use the Stryd power meter because it is widely available and has demonstrated high repeatability and a consistent relationship with $\dot{V}$O_2_ [27]. However, data from our study, and others [63], suggest the absolute power reported by the device may be overestimated compared with cycling power. In contrast, energy expenditure was considerably underestimated for kayaking when using TWD-kJ, which can be reconciled by the differences in gross efficiency across exercise modes. Mean gross efficiency values in our study were 20.0% for cycling, 10.1% for kayaking, and 26.2% for running (data not shown). As the models were trained using cyclists only, these differences in efficiency can explain both the large errors in predictions using TWD-kJ and the considerable improvements in prediction accuracy with model calibration (Table 4). From a practical perspective, people wishing to apply these models with the calibration adjustment can use the model coefficients from Table 3 to get a predicted value of carbohydrate or energy expenditure, then solve the equation calibration intercept + (predicted value × calibration slope) from Table 4. Taken together, our data support the use of prediction models (with sport-based calibration) to allow individuals to estimate their carbohydrate and energy expenditure based on commonly available, non-invasive measures.

In addition to measures of training load, other variables included in the prediction models were training load squared, type of training session (continuous or interval based), sex, session duration, $\dot{V}$O_2_ at VT_2_, $\dot{V}$O_2max_, and prior-day sRPE-TL. It is noteworthy that prior-day sRPE-TL appeared in all carbohydrate utilization models but none of the energy expenditure models (Table 3). Presumably, greater prior-day sRPE-TL values imply lower levels of starting muscle glycogen, which is known to shift substrate use away from carbohydrate and towards fat oxidation [34]. Practical application of the carbohydrate models may be challenged by people who do not use sRPE-TL; however this is among the easiest measures for someone to record, with the caveat that a validated scale is used [16]. A less optimal, yet potentially viable option for athletes who only use TSS would be to convert prior-day TSS values into estimated sRPE-TL using the regression equation − 65.4 + (TSS × 5.18). Details of this equation are provided in the ESM, which is based on the strong relationship (*r* = 0.86) between TSS and sRPE-TL observed in our data.

The use of squared training load terms are included in half of the models, allowing non-linear relationships to be modeled. The second ventilatory threshold was also relevant in nearly all models, either as $\dot{V}$O_2_ at VT_2_ or as a percentage of $\dot{V}$O_2max_, which ostensibly provides the same information when combined with $\dot{V}$O_2max_. This could be expected, as VT_2_ (also called the respiratory compensation point) represents the highest $\dot{V}$O_2_ (and therefore, energy expenditure) associated with steady-state lactate levels in the blood [64].

A strength of the study is the inclusion of male and female athletes across a range of fitness levels. Female athletes typically have a reduced RER during submaximal endurance exercise compared with male athletes, indicating lower relative carbohydrate and higher relative fat oxidation [65]. This is reflected in the model coefficients shown in Table 3. Although resting glycogen levels may vary in female individuals across the menstrual cycle [66], most studies have found no influence of menstrual phase on substrate oxidation during exercise, particularly in the fed state [67]. Therefore, despite the mechanistic rationale, we chose not to control for menstrual cycle phase in our female participants. For studies that have found an effect of menstrual phase on substrate oxidation, this has been observed during lower, but not moderate or higher intensity exercise [68]. For athletes taking oral contraceptive pills, it is possible that the active phase may be associated with increased fat oxidation during exercise than the inactive phase [69], but again most studies have found minimal effects [67] and so we did not control for this.

There are several limitations to this study that should be considered. First, we did not account for protein oxidation in our calculations. Measures of substrate oxidation during exercise are typically interpreted based on the assumption of negligible protein oxidation, but this assumption could be invalidated in the context of protein ingestion before or during exercise because of increased gluconeogenesis, which could decrease RER irrespective of any change in fat oxidation rate via transfer of the amino group to the urea cycle [4]. However, protein content was low in our standardized pre-exercise meal, which is itself a practical limitation of the study. We standardized the pre-exercise meal, which means it is likely that carbohydrate use will decrease when training in the fasted state, at least during lower intensity exercise [70], and increase following a higher carbohydrate pre-exercise meal [34]. Future studies could test the robustness of these models across different types of pre-exercise meals. In addition, these models may not be applicable to very short (< 15 min) or very long (> 90 min) exercise durations. The carbohydrate models are more likely to overestimate use during longer sessions, as there is an expected decrease in carbohydrate reliance as exercise duration extends [34]. Some error may have also been introduced in the TSS calculation, which uses functional threshold power (FTP) that is often established using 95% of the average power attained during a 20-min time trial [18]. To reduce participant burden, we used power at VT_2_ as an estimate of FTP. This approach has been used elsewhere [31], although direct comparisons have reported VT_2_ power as being both higher [71] and lower [72] than FTP. For cyclists applying our models using TSS, a corrected FTP based on fitness level could be recommended [73]. Although the use of FTP as a marker of intensity domains has been questioned [74], it could be considered that the best use of FTP may be in enabling an athlete to have a measure of TSS. Finally, it is possible that RPE-based training load values recorded in a laboratory setting may not be directly applicable to outdoor training, a finding observed in some [75] but not all [76] studies. Future studies should consider longer exercise durations (> 90 min), other exercise modalities such as rowing, and the influence of different pre-exercise nutritional intakes (e.g., fasted-state training vs pre-exercise carbohydrate or protein intake) and starting levels of muscle glycogen to further investigate the predictive capabilities of these models.

## Conclusions

This study presents a novel method for measuring and estimating both carbohydrate utilization and energy expenditure during endurance exercise using easily available measures of training load and laboratory-based testing. We found all measures of training load displayed very large correlations with both carbohydrate and energy expenditure during exercise, but TSS was the only measure of training load to accurately reflect similar total carbohydrate use between longer sessions at low-intensity interval training and shorter sessions at HIIT. Our prediction models can be effectively applied in running and kayaking when used with a calibration adjustment.

## Supplementary Information

Below is the link to the electronic supplementary material.

Supplementary file1 (PDF 379 KB)

## Declarations

### Availability of Data and Material

Data are available at https://osf.io/nqsu7/.

### Code Availability

Data and R code are available at https://osf.io/nqsu7/.
