# Supplementary material for: A Novel Method to Predict Carbohydrate and Energy Expenditure During Endurance Exercise Using Measures of Training Load
Source: Sports Med. 2024 Nov 1;55(3):753–74. doi: 10.1007/s40279-024-02131-z (PMC11985602; doi:10.1007/s40279-024-02131-z)
Supplement: Supplementary file 1 — Supplementary file1 (PDF 379 KB) [file 40279_2024_2131_MOESM1_ESM.pdf]

Supplemental Files

A Novel Method to Predict Carbohydrate and Energy Expenditure during Endurance Exercise Using Measures of Training Load

Authors: Jeffrey A. Rothschild[1,2*], Stuart Hofmeyr[2], Shaun J. McLaren[3,4], and Ed Maunder[2]

Affiliations:
[1] High Performance Sport New Zealand (HPSNZ), Auckland, New Zealand
[2] Sports Performance Research Institute New Zealand (SPRINZ), Auckland University of Technology, Auckland, New Zealand
[3] Newcastle Falcons Rugby Club, Newcastle upon Tyne, United Kingdom
[4] Department of Sport and Exercise Sciences, Manchester Metropolitan University Institute of Sport, Manchester, UK

*Corresponding author - Jeffrey Rothschild – Jeffrey.Rothschild@aut.ac.nz

Supplemental Table 1. Sample size calculations

	R2 apparent	Model parameters	R2 adjusted	Minimum sample size
**Kcal**				
TWD-kJ	0.96	3	0.95	7
TSS	0.96	3	0.95	7
TSS-HR	0.94	6	0.89	10
Lucia TRIMP power	0.93	4	0.90	11
Lucia TRIMP HR	0.93	5	0.89	11
sRPE-TL	0.93	5	0.89	11
**CHO**				
TSS-HR	0.93	5	0.89	11
TWD-kJ	0.92	4	0.89	13
TSS	0.92	6	0.86	13
Lucia TRIMP power	0.89	5	0.83	18
Lucia TRIMP HR	0.90	6	0.83	16
sRPE-TL	0.88	5	0.81	19

CHO = carbohydrate, R2 apparent = R2 from initial model, R2 adjusted = Adjusted R2 based on sample size and model parameters. Minimum sample size calculated as minimum data points required divided by two because each participant provides two data points.

Supplemental File 1

Examples from published literature

This approach to energy and carbohydrate calculations can be ‘stress-tested’ using previously published research which has measured muscle glycogen and/or other contributors to energy expenditure.

Assumptions

- To convert from kcal to grams of carbohydrate, consideration of the carbohydrate source is necessary, because the energy yield is 3.719 kcal per gram from glucose and 4.187 kcal per gram from glycogen (Livesey and Elia 1988).
- Glycogen was converted from dry weight to wet weight using the conversion factor (0.23) of Areta and Hopkins (2018), and to grams of carbohydrate assuming 45% muscle mass and 25% active muscle mass (Medbo and Tabata 1993). A molecular weight of glycogen of 162 was used in the conversion (Schaubroeck 2022). Conversion steps are shown as follows:
    1) Glycogen (mmol/kg wet weight) = Glycogen (mmol/kg wet weight) * 0.23
    2) Activated muscle mass = body mass (kg) * 0.45 * 0.25
    3) Glycogen (mmol) = mmol/kg wet weight * Activated muscle mass (kg)
    4) Glycogen (g) = Glycogen (mmol) * (162 / 1000)

Romijn et al (1993) used stable isotope tracers and indirect calorimetry were to evaluate the regulation of endogenous fat and glucose metabolism in relation to exercise intensity and duration.

Extracting Fig. 8 allows us to use these values in calculating estimated energy expenditure.

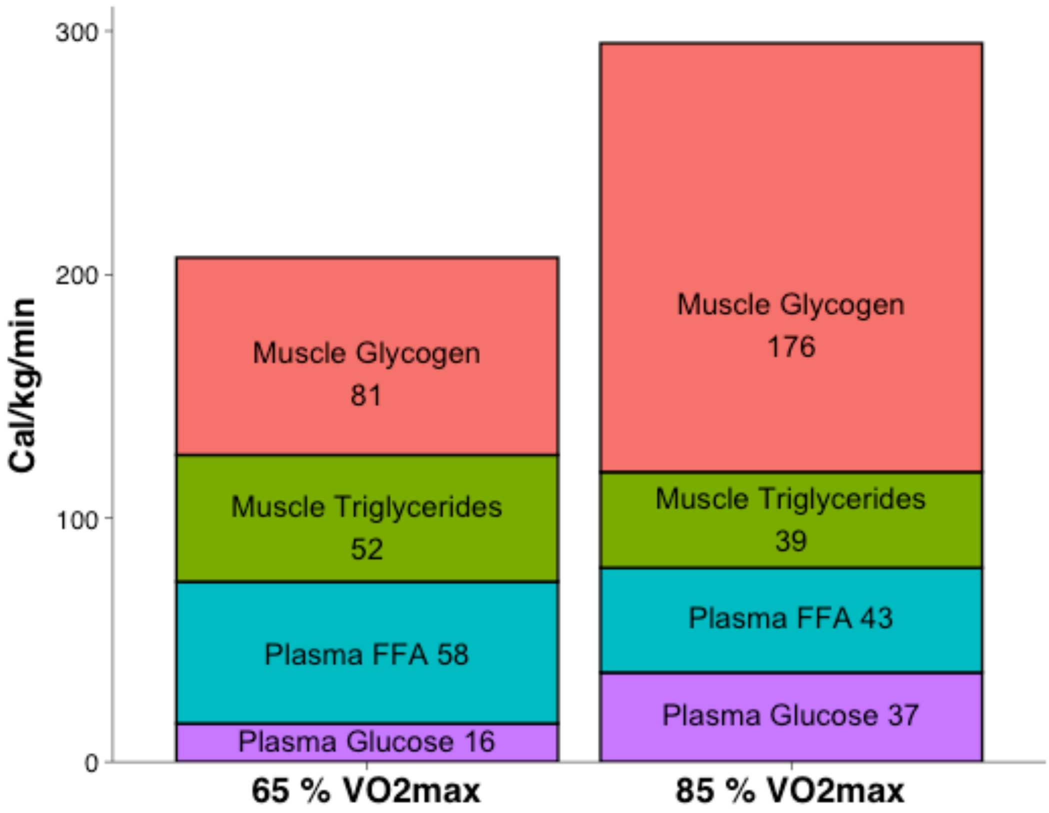

Extracted from Romijn et al (1993) Fig. 8

30-min at 85% VO_{2max}

Using data from Romijn et al (1993) we can estimate as follows based on the 30 min session at 85% VO_{2max}.

Source	Cal Kg Min	Body Weight	Kcal Min	Session Duration	Session Kcal
Plasma Glucose	36.7	75.2	2.76	30	82.8
Plasma FFA	43.0	75.2	3.23	30	96.9
Muscle Triglycerides	39.3	75.2	2.96	30	88.8
Muscle Glycogen	176.0	75.2	13.24	30	397.2

Summing everything together gives an estimate of **666 total kcal**, and summing together the contributions from glucose and glycogen after dividing by the appropriate yields of kcal/g from glucose (3.719) and glycogen (4.187), gives **117 g carbohydrate**.

We can also estimate the session expenditure using the summed aerobic (VO_2) + anaerobic (lactate) systems approach, assuming 3.77 mmol lactate accumulation during the 30 min of cycling (reported at 85% VO_{2max} in Romijn 1992), and an average RER value of 0.91.

```
romijn_delta_lactate <-  4.55- 0.78 # based on Romijn 1992 at 85% VO2max

romij_vo2_calc <- tibble(
    vo2_max = vo2max_romijn, # 5.04 L, reported in paper
    pct_max = .85,  # reported in paper
    vo2 = pct_max * vo2_max, # get absolute VO2
    rer = .91  # reported in paper
  ) %>%

  # get RER-adjusted VO2-kcal conversions from Elia and Livesey 1992
    left_join(kj_lookup_tbl, by = "rer") %>%

  # calculate kcal and carb for 1 minute
  mutate(
    kcal_from_vo2 = vo2 * kcal_L ,
    kcal_from_carb = pct_carb/100 * kcal_from_vo2,
    pct_from_glucose = 1- pct_max,  # intensity-adjusted
    yield_from_glucose = pct_from_glucose * glucose_yield, #glucose yields
3.719 kcal/g
    yield_from_glycogen = pct_max * glycogen_yield,  #glycogen yields 4.187
kcal/g
    glucose_glycogen_yield = yield_from_glucose + yield_from_glycogen,
    carb_ox_g_min = kcal_from_carb/glucose_glycogen_yield
  )
```

These calculations are reflected below:

	Value
vo2_max	5.04
pct_max	0.85
vo2	4.28
rer	0.91
kJ_L	20.65
kcal_L	4.94
pct_carb	70.53
kcal_from_vo2	21.14
kcal_from_carb	14.91
pct_from_glucose	0.15
yield_from_glucose	0.56
yield_from_glycogen	3.56
glucose_glycogen_yield	4.12
carb_ox_g_min	3.62

Multiplying the per-minute values by 30 and adding the anaerobic component:

```
romij_vo2_lactate_calc <- romij_vo2_calc %>%
  #multiply by 30 for 30 min session
  summarise(
    aerobic_carb_g = carb_ox_g_min * 30,
    aerobic_kcal = kcal_from_vo2 * 30
  ) %>%
  # calculate anaerobic energy expenditure using net lactate accumulation
method
  mutate(
    ml_o2_lactate = romijn_delta_lactate * 3 * bw_romijn,
    lactate_kj = ml_o2_lactate/1000 * 21.1,
     # kJ to kcal to grams * 11.845 to account for anaerobic inefficiency
    carb_from_lacate_g = lactate_kj/4.184/4.187  * 11.845,  #
    total_carb_g = round(carb_from_lacate_g + aerobic_carb_g,0),
    total_kcal = round(aerobic_kcal + (carb_from_lacate_g *
glycogen_yield),0),
    estimate = "Romijn VO2-lactate calculation",
  )
```

The above calculations result in the following values:

```
## # A tibble: 1 × 4
##   `Aerobic CHO (g)` `CHO (g) from lactate` `Aerobic kcal` `Total kcal`
##               <dbl>                  <dbl>          <dbl>        <dbl>
## 1               109                     12            634          685
```

When compared together they display good agreement.

Estimate	Total kcal	Total CHO (g)
Romijn additive calculation	666	117
Romijn VO2-lactate calculation	685	121

Wingate tests

We also calculated kcal and carbohydrate use from a study reporting muscle glycogen reductions during a single 30-s Wingate sprint (Esbjornsson-Liljedahl et al 1999), which also reported data for males and females separately.

We extracted VO_2 data from a separate study (Beneke et al 2002), adjusting for VO_{2max} to match each group

To estimate contributions from plasma glucose and muscle triglycerides we adapted the numbers from Romijn 1993, and used each group's reported values of muscle glycogen breakdown. Considering both the intensity and short duration, FFA contribution was deemed negligible.

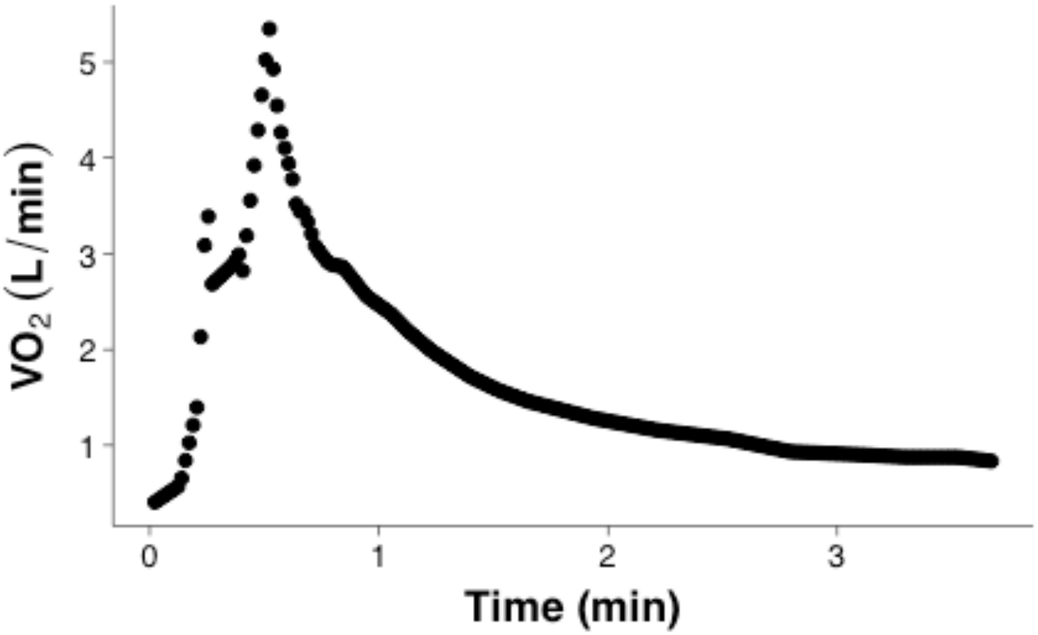

Extracted from Beneke at al (2002) Fig. 1

This study also reported fiber-type specific glycogen reduction, which was then adjusted based on relative area of fiber types reported in Table 1 of the paper. We assumed for average RER value of 1.0 during the 3.5 min window.

```
esbj_session_duration <- 3.5
wingate_glucose_cal_kg_min <- 36.7
wingate_tg_cal_kg_min <- 39.3
wingate_avg_rer <- 1
beneke_vo2max <- 4.22

Esbj_additive_calcs <- tibble(
  sex = c("m", "f"),
  mass = c(75, 65),
  session_duration = esbj_session_duration,
  fat_cal_min = wingate_tg_cal_kg_min,
  fat_kcal = fat_cal_min * mass * session_duration/1000,
  glucose_cal_min =  wingate_glucose_cal_kg_min,
  glucose_kcal = glucose_cal_min * mass * session_duration/1000,
  glucose_g = glucose_kcal/ glucose_yield,
  quad_mass = mass * .45 * .25,
  type1pct = c(56, 66)/100,
  type2pct = 1-type1pct,
  glycogen_delta_type1 = c((452-326), 428-355),
  glycogen_delta_type2 = c((526-395), (542-393)),
  weighted_glycogen_delta_dm = (glycogen_delta_type1 * type1pct +
glycogen_delta_type2 * type2pct),
```

```
  weighted_glycogen_delta_wm = weighted_glycogen_delta_dm * .23,
  glycogen_g = convert_glycogen_to_grams(weighted_glycogen_delta_wm,
quad_mass),
  glycogen_kcal = glycogen_g * glycogen_yield,
  total_kcal = fat_kcal + glucose_kcal + glycogen_kcal,
  total_carb_g = glycogen_g + glucose_g,
  estimate = c("Esbjornsson additive calculation - males", "Esbjornsson
additive calculation - females"),
)
```

These calculations result in the following values:

Calculation	M	F
mass	75.00	65.00
session_duration	3.50	3.50
fat_cal_min	39.30	39.30
fat_kcal	10.32	8.94
glucose_cal_min	36.70	36.70
glucose_kcal	9.63	8.35
glucose_g	2.59	2.25
quad_mass	8.44	7.31
type1pct	0.56	0.66
type2pct	0.44	0.34
glycogen_delta_type1	126.00	73.00
glycogen_delta_type2	131.00	149.00
weighted_glycogen_delta_dm	128.20	98.84
weighted_glycogen_delta_wm	29.49	22.73
glycogen_g	40.30	26.93
glycogen_kcal	168.75	112.76
total_kcal	188.70	130.05
total_carb_g	42.89	29.18

```
Esbj_lactate_calcs <- tibble(
  sex = c("m", "f"),
  mass = c(75, 65),
  lactate_delta = c(11.8- 1.46, 9.35-1.35),
  ml_o2_lactate = 3 * lactate_delta * mass,
  lactate_kj = ml_o2_lactate/1000 * 21.1,
  carb_from_lacate_g = round(lactate_kj/4.184/4.187  * 11.845,1)
)

Esbj_lactate_calcs

## # A tibble: 2 × 6
##   sex    mass lactate_delta ml_o2_lactate lactate_kj carb_from_lacate_g
```

```
##   <chr> <dbl>          <dbl>         <dbl>      <dbl>                <dbl>
## 1 m        75           10.3         2326.       49.1                 33.2
## 2 f        65            8           1560        32.9                 22.3

# Adjust VO2 values for each group's VO2max
Esbj_vo2max_tbl <- tibble(
  sex = c("m", "f"),
  mass = c(75, 65),
  est_vo2max = c(55, 45),
  vo2max_l = est_vo2max * mass/ 1000,
  adjustment_factor_from_beneke = vo2max_l/ beneke_vo2max  # adjust for
group-specific VO2max values
)


vo2_calcs_tbl <- wingate_df %>%
  interpolate() %>%
  mutate(
    m = vo2 * Esbj_vo2max_tbl$adjustment_factor_from_beneke[1],
    f = vo2 * Esbj_vo2max_tbl$adjustment_factor_from_beneke[2],
    rer = wingate_avg_rer
  ) %>% select(-vo2) %>%

  pivot_longer(m:f, names_to = "sex", values_to = "vo2") %>%
  nest(.by = sex) %>%
  left_join(Esbj_vo2max_tbl %>% select(sex, vo2max_l), by = "sex") %>%
  unnest(data) %>%
  mutate(pct_max = vo2/vo2max_l) %>%
  nest(.by = sex) %>%
  mutate(
    # apply second-by-second aerobic energy calculations function
    totals = map(data, calculate_carb_kcal_fn)
  ) %>% unnest(totals) %>% select(-data)  %>%
  left_join(Esbj_lactate_calcs, by = "sex") %>%
  mutate(
    estimate = c("Esbjornsson VO2-lactate calculation - males", "Esbjornsson
VO2-lactate calculation - females"),
    total_kcal = aerobic_kcal + carb_from_lacate_g * glycogen_yield,
    total_carb_g = aerobic_carb_g + carb_from_lacate_g, .keep = "unused"
  ) %>% select(estimate, total_kcal, total_carb_g)
```

Good agreement is seen for both males and females

Estimate	Total kcal	Total CHO (g)
Esbjornsson additive calculation - males	189	43
Esbjornsson VO2-lactate calculation - males	169	41
Estimate	Total kcal	Total CHO (g)
Esbjornsson additive calculation - females	130	29
Esbjornsson VO2-lactate calculation - females	115	28

Supplemental File 2

Converting TSS to sRPE-TL

For people wishing to use the carbohydrate models who don't have access to prior-day sRPE-TL data, prior-day TSS could be substituted by using a conversion to estimated sRPE-TL (albeit with some acknowledged trade-offs in terms of accuracy).

Using data combined from the primary and validation studies, there is a strong correlation (r = 0.86) between TSS and sRPE-TL. Therefore, a regression line can be estimated and used to convert prior day TSS to a value of sRPE-TL which can then be used in the prediction models.

```
tss_convert1 <- lmer(sRPE_TL ~ TSS + (1 | subject_id), mod_refit_tbl)

tss_convert1

## Linear mixed model fit by REML ['lmerModLmerTest']
## Formula: sRPE_TL ~ TSS + (1 | subject_id)
##    Data: mod_refit_tbl
## REML criterion at convergence: 1973.78
## Random effects:
##  Groups     Name        Std.Dev.
##  subject_id (Intercept) 41.56
##  Residual               75.91
## Number of obs: 169, groups:  subject_id, 70
## Fixed Effects:
## (Intercept)          TSS
##     -65.389        5.177
```

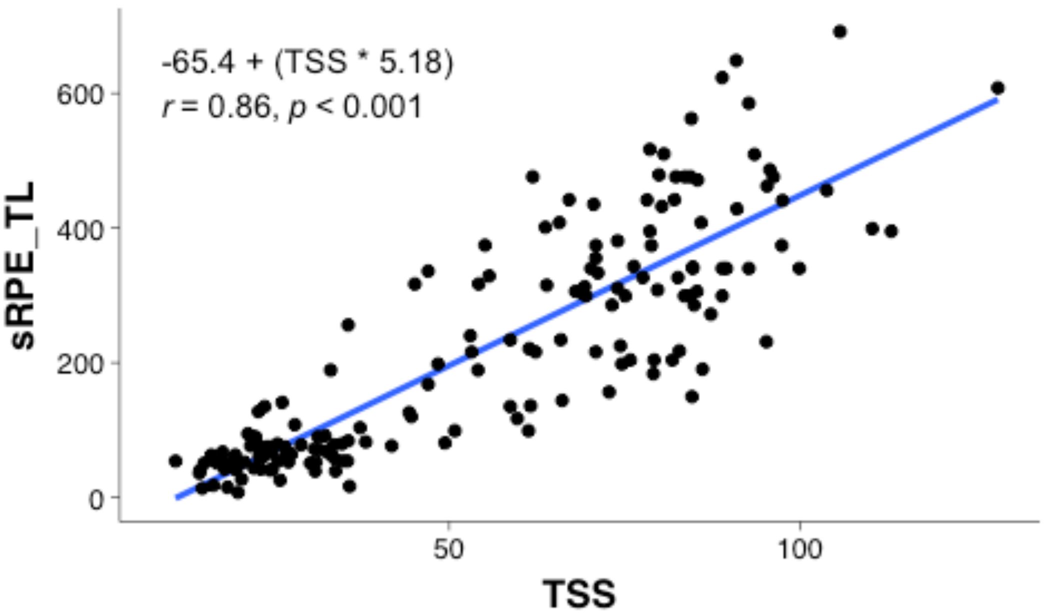

So for example if you have a TSS of 100, use the equatoion -65.4 + (TSS * 5.18) to estimate a value of 453.
